# Decomposing the change of suicide rates in the United States 2001–2023

**DOI:** 10.1017/S2045796025100218

**Published:** 2025-09-04

**Authors:** Paul S.F. Yip, Yu Cheng Hsu, Tsz Mei Lam, Yunyu Xiao, Eric Caine

**Affiliations:** 1 The Hong Kong Jockey Club Centre for Suicide Research and Prevention, Faculty of Social Sciences, The University of Hong Kong, Hong Kong, China; 2 Department of Social Work and Social Administration, Faculty of Social Sciences, The University of Hong Kong, Hong Kong, China; 3 Department of Population Health Sciences, Weill Cornell Medicine, New York Presbyterian, New York, NY, USA; 4 Department of Psychiatry, Weill Cornell Medicine, New York Presbyterian, New York, NY, USA; 5 University of Rochester Medical Center, Rochester, NY, USA

**Keywords:** decomposition analysis, epidemiology, firearm, observational study, suicide

## Abstract

**Introduction:**

Suicide rates in the United States have been increasing, necessitating an understanding of demographic variations by ethnicity, age, sex and method to inform effective prevention strategies.

**Objective:**

To dissect suicide rates in the US population from 2001 to 2023 by age, sex, ethnicity, and method.

**Methods:**

This retrospective observational study utilized suicide data and population statistics from the CDC’s WISQARS database for the years 2001 (*n* = 30,418), 2018 (*n* = 48,132), 2020 (*n* = 45,721) and 2023 (*n* = 49,014). Cases were stratified by age, sex, ethnicity, and suicide method to assess trends and demographic differences.

**Results:**

From 2001 to 2023, the overall US suicide rate rose from 10.7 to 14.6 per 100,000, with a temporary decrease in 2019 and 2020 (14.4 and 13.8, respectively). The primary driver of the increase was firearm-related suicides among White males, contributing 25.8% of the rise from 2001 to 2018 and 51.6% from 2020 to 2023. Decline between 2018 and 2020 was mainly due to reductions in firearm and drug-related suicides among White males, but firearm suicides surged again from 2020 to 2023. Additionally, firearm suicides among ethnic minorities, especially Black/African-American males, accounted for 14.0% of the increase during 2020–2023. Drug-related suicides also increased by 8.6% among White females aged 45 and older in the same period.

**Conclusions:**

Firearm suicides are the leading factor in the changing suicide rates in the United States from 2001 to 2023, alongside rising drug-related suicides among White females. These trends highlight the necessity for targeted prevention efforts that consider demographic-specific factors and method accessibility.

## Introduction

Suicide remains a significant global public health concern (WHO, 2024). In the United States, suicide rates had increased steadily from 2001 through 2018 (Hedegaard and Warner, 2021), dropping briefly and then increasing again. Understanding the complex interplay of factors contributing to these changes is crucial for developing effective prevention strategies. This study aims to provide a comprehensive analysis of suicide trends in the United States from 2001 to 2023, focusing on the change in suicide rates by method, ethnicity, age, and sex.

The overall US suicide rates increased from 2000 to 2018, declined from 2018 to 2020, and again increased. Existing studies have discussed the differences in suicide rates by methods, ethnicity, age groups, and sex (Curtin *et al.,* 2022; Dhungel *et al.,* 2024; Hedegaard and Warner, 2021; Karaye, 2022; Ramchand *et al.,* 2021). Nonetheless, existing research works tend to marginalize at least one of the demographic variables for the analysis. For example, Hedegaard and Warner (2021) neglected age in analysing the suicide rate differences among different suicide methods and neglected means of suicide in analysing the suicide rate differences among various age groups. This could make the prevention measures less targeted to the population in need and might cause ineffective use of resources.

Previous research has identified various risk factors for suicide, including mental health disorders (De La Garza *et al.,* 2021; Hoertel *et al.,* 2015; Steele *et al.,* 2018), substance abuse (Poorolajal *et al.,* 2016; Steele *et al.,* 2018), social isolation (Motillon-Toudic *et al.,* 2022; Steele *et al.,* 2018) and access to lethal means (Hawton *et al.,* 2024; Yip *et al.,* 2012). However, the relative contribution of these factors to overall suicide rates and how they vary across different demographic groups remains unclear. In addition, between 2001 and 2023, there were significant societal changes, including the aftermath of the 2008 financial crisis, the extraordinary increase in opioid-related fatalities and the COVID-19 pandemic (Pirkis *et al.,* 2022). This study first used joinpoint regression to identify statistically significant change points of the US suicide rates that make a sign change, i.e., from increasing to decreasing or from decreasing to increasing. Based on the identified key time points (2001, 2018, 2020, and 2023), this study aims to describe which populations contributed to significant changes (decrease/increase) in suicide rates. Furthermore, understanding the relative contribution of firearms (the most commonly used fatal method) and drug ingestion (the most common method for attempts) to overall suicide rates can inform targeted prevention strategies and policy interventions (Cai *et al.,* 2021). By employing a decomposition analysis, this study seeks to elucidate the specific contributions of suicide methods, population structure changes and suicide rate changes within different demographic strata (Yang and Yip, 2021; Yip *et al.,* 2022).

## Methods

### Data for suicide and population

The suicide cases count stratified by age group, sex, ethnicity, and methods were retrieved from the nationally representative US Centers for Disease Control and Prevention (CDC) annual mortality data files for Web-based Injury Statistics Query and Reporting System (WISQARS) Fatal data (2025).

For the analysis between 2001 and 2018, we used the data from the bridged race dataset, with 30,418 and 48,132 suicide decedents of all ages respectively. For the comparison among the following 3 years: 2018, 2020, and 2023 single race database was used, which had 48,074, 45,721, and 49,014 suicide decedents of all ages, respectively. Data were accessed on May 10, 2025. The population data for each stratum were collected from the WISQARS Fatal data as well.

### Measures

This study decomposed the suicide rate by suicide method, ethnicity, age groups, and sex-specific number of suicides in all 50 states and the District of Columbia. In this study, the suicide method was categorized into three groups based on the International Classification of Diseases version 10 codes (ICD-10): Firearm suicides (X72-X74), drug-related suicides (X60–X64), suffocation (X70) and non-firearm, non-drug suicides and non-suffocation (X65–X69, X71, X75–X84), which is obtained through all suicide caused deducted by the firearm suicide cases, drug suicide cases and suffocation cases. We did not collect data for Y87.0, sequelae of intentional self-harm, as this classification does not specify the method used. In 2001, ethnicity was first divided into Hispanic and non-Hispanic, and then by race, including (non-Hispanic): American Indian/Alaska Native, Asian/Native Hawaiian/Pacific Islanders, Black/African American, and White. In the years 2018, 2020 and 2023, ethnicity was divided into Hispanic and non-Hispanic, and then by race, including (non-Hispanic): American Indian/Alaska Native, Asian, Black/African American, Native Hawaiian (HI)/Other Pacific Islander, White and More than one race (who indicate more than one race). Age was classified as children (below 15), young (15–24 years), younger middle-aged (25–44 years), older middle-aged (45–64 years) and older adults (65 or above) as they have shown distinct patterns. Likewise, sex, male and female, was also included.

### Data analysis

Turning points of the overall US suicide rate temporal trend were detected through joinpoints regression (Kim *et al.,* 2000). These turning points were used to determine the time in the decompositional analysis. The joinpoint regression was configured with the constraints that there is at least an observation between two change points and assumed constant and uncorrelated residuals. The optimal number of turning points and location were determined by the BIC criteria.

Decompositional analysis (Kitigawa, 1955; Preston *et al.,* 2001) was employed to examine the contribution of different factors to changes in suicide rates in each time segment. This method has been widely used in disentangling demographic and rate changes involving suicide (Yang and Yip, 2021; Yip *et al.,* 2022). The computational methods for decompositional analyses are provided in eMethods. The current decompositional analysis considered the rate changes of suicide between the start and end years of each segment, breaking down the overall effect into the population structure changes and suicide rate changes in different demographic strata, including ethnicity, age groups and sex, as well as the method of suicide. This approach facilitated a better understanding of the factors underlying the observed patterns.

As the WISQARS fatal data does not provide the amount of decedent number for the stratum with less than 10 cases, these strata are assumed to be zero to ensure the decompositional analysis formula works.

## Results

### Overall suicide trends

**Figure 1.**
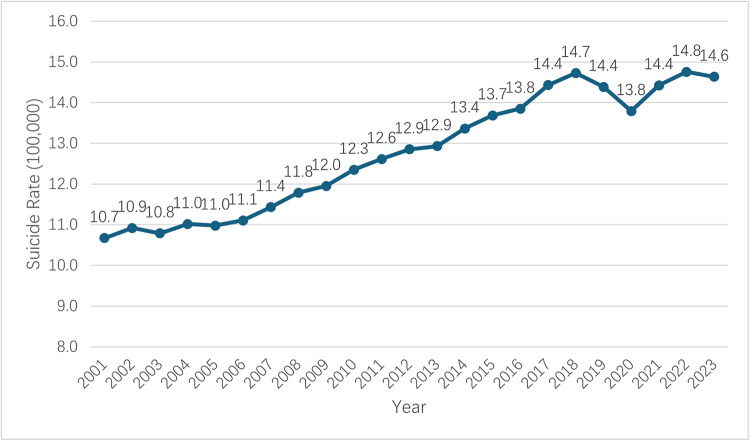
Crude suicide rate per 100,000 persons in the US from 2001 to 2023.

**Figure 2.**
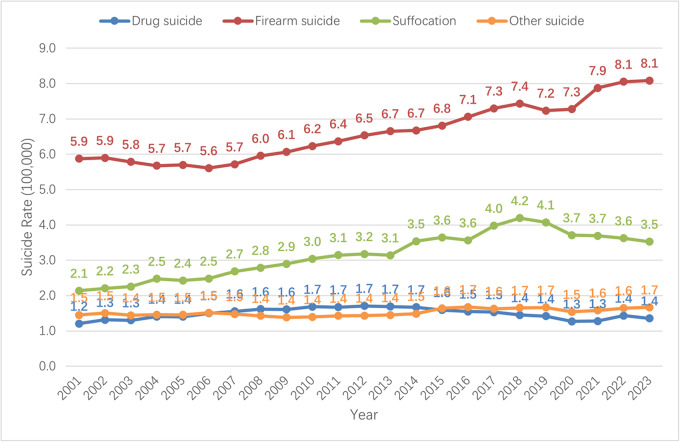
Methods-specific crude suicide rate per 100,000 persons in the US from 2001 to 2023.

The overall suicide rates increased from 2001 to 2023, with the major increment coming from suicides caused by firearms. Figure 1 reveals the crude suicide rate change in the United States. In addition to an overall increasing trend during the period, a slight drop in the suicide rate from 2018 to 2020 was observed and then increased again after 2020. Figure 2 reveals the methods-specific crude suicide rate in the United States. Echoing the trend in Fig. 1, the trends of method-specific suicide rates show that firearm suicide increased from 5.9 per 100,000 people in 2001 to 8.1 per 100,000 people in 2023. Suffocation suicide methods (e.g., hanging) rose from 2.1 per 100,000 in 2001 to the peak 4.2 per 100,000 in 2018 and decreased to 3.5 per 100,000 people in 2023. Drug-related suicide slowly increased from 1.2 per 100,000 people in 2001 to 1.4 per 100,000 people in 2023.

### Decomposition of suicide rates in the three-time segments

The decomposition analysis of suicide rate trends in the United States in 2001, 2018, 2020 and 2023 revealed insights into the relative increment of different population structures and suicide methods, particularly for drugs and firearms.

During the period from 2001 to 2018, firearm and suffocation suicide among the White male population accounted for 25.8% and 27.9% of the total suicide increase from 2001 to 2018, as in Table 1. While evident in all age strata, the increase was most prominent for those aged 45–64 years. A parallel trend was observed among the White female population. In addition, Table 1 also shows the increase in drug suicide among females for 0.21 per 100,000 people, especially those White female at 45–64 and 65 or above age group, which counted one-third of the increment of drug-related suicides during this period.

**Table 1.** Contribution of age, sex, ethnicity and methods to the increase of the US suicide rate during the period 2001–2018

	Population structure, suicide rate per 100,000 people (relative contribution %)				
	American Indian or Alaska Native	Asian/Native Hawaiian/Pacific Islanders	Black or African American	Hispanic	White
**Male**					
Below 15	0.0000 (0.00%)	0.0000 (0.00%)	−0.0010 (−0.02%)	0.0019 (0.05%)	−0.0131 (−0.32%)
15−24	−0.0024 (−0.06%)	0.0060 (0.15%)	−0.0005 (−0.01%)	0.0209 (0.51%)	−0.1756 (−4.32%)
25−44	−0.0021 (−0.05%)	0.0207 (0.51%)	−0.0169 (−0.42%)	0.0701 (1.72%)	−0.6632 (−16.31%)
45−64	0.0033 (0.08%)	0.0188 (0.46%)	0.0258 (0.64%)	0.1013 (2.49%)	−0.0473 (−1.16%)
65 or above	0.0000 (0.00%)	0.0260 (0.64%)	0.0055 (0.13%)	0.0569 (1.40%)	0.4231 (10.41%)
**Subtotal**	−0.0012 (−0.03%)	0.0715 (1.76%)	0.0129 (0.32%)	0.2511 (6.17%)	−0.4761 (−11.71%)
**Female**					
Below 15	0.0000 (0.00%)	0.0000 (0.00%)	0.0000 (0.00%)	0.0009 (0.02%)	−0.0039 (−0.10%)
15−24	−0.0006 (−0.02%)	0.0015 (0.04%)	−0.0004 (−0.01%)	0.0079 (0.20%)	−0.0298 (−0.73%)
25−44	−0.0007 (−0.02%)	0.0069 (0.17%)	−0.0040 (−0.10%)	0.0123 (0.30%)	−0.1811 (−4.45%)
45−64	0.0000 (0.00%)	0.0106 (0.26%)	0.0121 (0.30%)	0.0190 (0.47%)	−0.0191 (−0.47%)
65 or above	0.0000 (0.00%)	0.0092 (0.23%)	0.0033 (0.08%)	0.0046 (0.11%)	0.0273 (0.67%)
**Subtotal**	−0.0013 (−0.03%)	0.0281 (0.69%)	0.0110 (0.27%)	0.0448 (1.10%)	−0.2066 (−5.08%)
	**American Indian or Alaska Native, suicide rate per 100,000 people (relative contribution %)**				
	**Drug suicide**	**Firearm suicide**	**Suffocation**	**Other suicide**	
**Male**					
Below 15	0.0000 (0.00%)	0.0000 (0.00%)	0.0052 (0.13%)	0.0000 (0.00%)	
15−24	0.0000 (0.00%)	0.0050 (0.12%)	0.0067 (0.17%)	0.0015 (0.04%)	
25−44	0.0000 (0.00%)	0.0053 (0.13%)	0.0110 (0.27%)	0.0014 (0.03%)	
45−64	0.0031 (0.08%)	0.0033 (0.08%)	0.0000 (0.00%)	−0.0035 (−0.09%)	
65 or above	0.0000 (0.00%)	0.0061 (0.15%)	0.0000 (0.00%)	0.0012 (0.03%)	
**Subtotal**	0.0031 (0.08%)	0.0198 (0.49%)	0.0229 (0.56%)	0.0007 (0.02%)	
**Female**					
Below 15	0.0000 (0.00%)	0.0000 (0.00%)	0.0052 (0.13%)	0.0003 (0.01%)	
15−24	0.0000 (0.00%)	0.0000 (0.00%)	0.0067 (0.17%)	−0.0017 (−0.04%)	
25−44	−0.0042 (−0.10%)	0.0052 (0.13%)	0.0110 (0.27%)	−0.0009 (−0.02%)	
45−64	0.0034 (0.08%)	0.0000 (0.00%)	0.0000 (0.00%)	0.0052 (0.13%)	
65 or above	0.0000 (0.00%)	0.0000 (0.00%)	0.0000 (0.00%)	0.0000 (0.00%)	
**Subtotal**	−0.0009 (−0.02%)	0.0052 (0.13%)	0.0229 (0.56%)	0.0030 (0.07%)	
	**Asian/Native Hawaiian/Pacific Islanders, suicide rate per 100,000 people (relative contribution %)**				
	**Drug suicide**	**Firearm suicide**	**Suffocation**	**Other suicide**	
**Male**					
Below 15	0.0000 (0.00%)	0.0000 (0.00%)	0.0043 (0.11%)	0.0021 (0.05%)	
15−24	0.0034 (0.08%)	0.0026 (0.06%)	0.0165 (0.41%)	0.0085 (0.21%)	
25−44	0.0012 (0.03%)	0.0055 (0.13%)	0.0124 (0.31%)	0.0121 (0.30%)	
45−64	0.0043 (0.11%)	0.0080 (0.20%)	0.0203 (0.50%)	0.0034 (0.08%)	
65 or above	0.0000 (0.00%)	−0.0045 (−0.11%)	−0.0029 (−0.07%)	0.0012 (0.03%)	
**Subtotal**	0.0089 (0.22%)	0.0115 (0.28%)	0.0505 (1.42%)	0.0274 (0.67%)	
**Female**					
Below 15	0.0000 (0.00%)	0.0000 (0.00%)	0.0000 (0.00%)	0.0000 (0.00%)	
15−24	0.0031 (0.08%)	0.0043 (0.11%)	0.0094 (0.23%)	−0.0007 (−0.02%)	
25−44	0.0013 (0.03%)	0.0020 (0.05%)	0.0111 (0.27%)	0.0031 (0.08%)	
45−64	0.0073 (0.18%)	−0.0012 (−0.03%)	0.0047 (0.11%)	0.0011 (0.03%)	
65 or above	0.0000 (0.00%)	0.0000 (0.00%)	−0.0053 (−0.13%)	0.0026 (0.06%)	
**Subtotal**	0.0117 (0.29%)	0.0050 (0.12%)	0.0199 (0.61%)	0.0061 (0.15%)	
	**Black/African-American, suicide rate per 100,000 people (relative contribution %)**				
	**Drug suicide**	**Firearm suicide**	**Suffocation**	**Other suicide**	
**Male**					
Below 15	0.0000 (0.00%)	0.0061 (0.15%)	0.0092 (0.23%)	−0.0021 (−0.05%)	
15−24	0.0046 (0.11%)	0.0056 (0.14%)	0.0305 (0.75%)	0.0060 (0.15%)	
25− 44	0.0024 (0.06%)	0.0597 (1.47%)	0.0609 (1.50%)	0.0144 (0.35%)	
45 −64	0.0015 (0.04%)	0.0193 (0.47%)	0.0160 (0.39%)	−0.0045 (−0.11%)	
65 or above	0.0000 (0.00%)	−0.0002 (0.00%)	−0.0013 (−0.03%)	0.0039 (0.10%)	
**Subtotal**	0.0086 (0.21%)	0.0906 (2.23%)	0.1154 (2.97%)	0.0178 (0.44%)	
**Female**					
Below 15	0.0000 (0.00%)	0.0000 (0.00%)	0.0049 (0.12%)	0.0024 (0.06%)	
15−24	0.0040 (0.10%)	0.0067 (0.17%)	0.0150 (0.37%)	0.0037 (0.09%)	
25−44	0.0018 (0.04%)	0.0086 (0.21%)	0.0184 (0.45%)	0.0083 (0.20%)	
45−64	0.0022 (0.05%)	0.0010 (0.02%)	−0.0066 (−0.16%)	−0.0039 (−0.10%)	
65 or above	0.0000 (0.00%)	−0.0025 (−0.06%)	−0.0059 (−0.15%)	0.0020 (0.05%)	
**Subtotal**	0.0080 (0.20%)	0.0139 (0.34%)	0.0258 (0.72%)	0.0126 (0.31%)	
	**Hispanic, suicide rate per 100,000 people (relative contribution %)**				
	**Drug suicide**	**Firearm suicide**	**Suffocation**	**Other suicide**	
**Male**					
Below 15	0.0000 (0.00%)	0.0046 (0.11%)	0.0034 (0.08%)	−0.0015 (−0.04%)	
15−24	0.0055 (0.14%)	0.0433 (1.06%)	0.0446 (1.10%)	0.0157 (0.39%)	
25−44	−0.0026 (−0.06%)	0.0260 (0.64%)	0.0773 (1.90%)	0.0136 (0.34%)	
45−64	0.0055 (0.14%)	−0.0055 (−0.13%)	0.0439 (1.08%)	0.0050 (0.12%)	
65 or above	0.0058 (0.14%)	−0.0123 (−0.30%)	−0.0090 (−0.22%)	−0.0036 (−0.09%)	
**Subtotal**	0.0143 (0.39%)	0.0561 (2.12%)	0.1603 (4.17%)	0.0292 (0.93%)	
**Female**					
Below 15	0.0000 (0.00%)	0.0000 (0.00%)	0.0061 (0.15%)	0.0005 (0.01%)	
15−24	0.0073 (0.18%)	0.0037 (0.09%)	0.0144 (0.35%)	−0.0001 (0.00%)	
25−44	0.0056 (0.14%)	0.0098 (0.24%)	0.0323 (0.80%)	0.0115 (0.28%)	
45−64	0.0030 (0.07%)	0.0003 (0.01%)	0.0226 (0.56%)	−0.0074 (−0.18%)	
65 or above	0.0049 (0.12%)	0.0000 (0.00%)	0.0031 (0.08%)	−0.0046 (−0.11%)	
**Subtotal**	0.0208 (0.57%)	0.0139 (0.35%)	0.0785 (1.85%)	−0.0001 (0.13%)	
	**White, suicide rate per 100,000 people (relative contribution %)**				**Overall, suicide rate per 100,000 people (relative contribution %)**
	**Drug suicide**	**Firearm suicide**	**Suffocation**	**Other suicide**	
**Male**					
Below 15	0.0000 (0.00%)	0.0204 (0.50%)	0.0149 (0.37%)	0.0009 (0.02%)	0.0555 (1.36%)
15−24	0.0030 (0.07%)	0.1521 (3.74%)	0.0939 (2.31%)	0.0399 (0.98%)	0.3374 (8.30%)
25−44	−0.0595 (−1.46%)	0.2492 (6.13%)	0.4941 (12.15%)	0.0566 (1.39%)	0.4497 (11.06%)
45−64	0.0600 (1.48%)	0.4936 (12.14%)	0.5163 (12.70%)	0.0900 (2.21%)	1.3821 (33.99%)
65 or above	0.0437 (1.07%)	0.1318 (3.24%)	0.0431 (1.06%)	−0.0036 (−0.09%)	0.7110 (17.49%)
**Subtotal**	0.0472 (1.16%)	1.0472 (25.75%)	1.1623 (27.85%)	0.1839 (4.52%)	2.9357 (72.19%)
**Female**					
Below 15	0.0037 (0.09%)	0.0070 (0.17%)	0.0172 (0.42%)	−0.0004 (−0.01%)	0.0440 (1.08%)
15−24	0.0126 (0.31%)	0.0292 (0.72%)	0.0642 (1.58%)	0.0146 (0.36%)	0.1751 (4.31%)
25−44	−0.0007 (−0.02%)	0.0658 (1.62%)	0.1871 (4.60%)	0.0154 (0.38%)	0.2259 (5.56%)
45−64	0.1035 (2.55%)	0.1350 (3.32%)	0.1807 (4.44%)	0.0332 (0.82%)	0.5066 (12.46%)
65 or above	0.0906 (2.23%)	0.0485 (1.19%)	0.0101 (0.25%)	−0.0087 (−0.21%)	0.1792 (4.41%)
**Subtotal**	0.2096 (5.16%)	0.2855 (7.02%)	0.4593 (11.25%)	0.0542 (1.33%)	1.1307 (27.81%)

Some cells are assumed to be 0.0000 (0.00%) due to the CDC WONDER would not provide strata with value less than 10 cases.

From the period between 2018 and 2020, there was a drop in the suicide rate – primarily due to a decrease in the suicide rate among the White population. Table 2 presents the decline in the US suicide rate during 2018–2020, with the 45–64 age male accounting for 55.9% of this decrease. The White population saw a decline for all suicide methods, particularly among those aged 45–64 (firearm: 19.1% in males and 5.8% in females; suffocation: 14.7% in males and 6.0% in females).

**Table 2.** Contribution of age, sex, ethnicity and methods to the increase of the US suicide rate during the period 2018–2020

	Population structure, suicide rate per 100,000 people (relative contribution %)						
	American Indian or Alaska Native	Asian/Native Hawaiian/Pacific Islanders	Black or African American	HI Native/Pacific Islander	Hispanic	White	More than one race
**Male**							
Below 15	0.0000 (0.00%)	0.0001 (−0.01%)	0.0000 (0.00%)	0.0000 (0.00%)	0.0002 (−0.02%)	−0.0014 (0.15%)	0.0001 (−0.01%)
15−24	−0.0014 (0.14%)	0.0000 (0.00%)	−0.0062 (0.64%)	0.0000 (0.00%)	0.0055 (−0.56%)	−0.0264 (2.71%)	0.0017 (−0.18%)
25−44	0.0001 (−0.01%)	0.0022 (−0.23%)	0.0075 (−0.77%)	0.0002 (−0.03%)	0.0095 (−0.98%)	−0.0098 (1.01%)	0.0041 (−0.42%)
45−64	−0.0003 (0.03%)	0.0052 (−0.53%)	0.0018 (−0.18%)	0.0000 (0.00%)	0.0211 (−2.16%)	−0.0671 (6.87%)	0.0007 (−0.07%)
65 or above	0.0004 (−0.04%)	0.0037 (−0.38%)	0.0035 (−0.36%)	0.0000 (0.00%)	0.0064 (−0.66%)	0.0542 (−5.54%)	0.0008 (−0.08%)
**Subtotal**	−0.0011 (0.12%)	0.0112 (−1.15%)	0.0065 (−0.67%)	0.0002 (−0.02%)	0.0428 (−4.38%)	−0.0507 (5.18%)	0.0075 (−0.76%)
**Female**							
Below 15	−0.0002 (0.03%)	0.0000 (0.00%)	0.0000 (0.00%)	0.0000 (0.00%)	0.0002 (−0.02%)	−0.0009 (0.09%)	0.0000 (0.00%)
15−24	−0.0003 (0.03%)	0.0001 (−0.02%)	−0.0011 (0.11%)	0.0000 (0.00%)	0.0018 (−0.19%)	−0.0058 (0.59%)	0.0005 (−0.05%)
25−44	−0.0001 (0.01%)	0.0003 (−0.03%)	0.0010 (−0.10%)	0.0000 (0.00%)	0.0025 (−0.26%)	−0.0078 (0.80%)	0.0011 (−0.11%)
45−64	−0.0003 (0.03%)	0.0012 (−0.13%)	−0.0006 (0.06%)	0.0000 (0.00%)	0.0037 (−0.38%)	−0.0516 (5.28%)	0.0003 (−0.03%)
65 or above	0.0000 (0.00%)	0.0010 (−0.11%)	0.0005 (−0.05%)	0.0000 (0.00%)	0.0011 (−0.11%)	0.0003 (−0.03%)	0.0000 (0.00%)
**subtotal**	−0.0009 (0.09%)	0.0027 (−0.28%)	−0.0002 (0.02%)	0.0000 (0.00%)	0.0092 (−0.95%)	−0.0658 (6.73%)	0.0018 (−0.19%)
	**American Indian or Alaska Native, suicide rate per 100,000 people (relative contribution %)**						
	**Drug suicide**	**Firearm suicide**	**Suffocation**	**Other suicide**			
**Male**							
Below 15	0.0000 (0.00%)	0.0000 (0.00%)	0.0000 (0.00%)	0.0039 (−0.40%)			
15−24	0.0000 (0.00%)	0.0060 (−0.62%)	−0.0043 (0.44%)	0.0007 (−0.07%)			
25−44	0.0000 (0.00%)	0.0030 (−0.31%)	−0.0033 (0.34%)	0.0020 (−0.21%)			
45−64	0.0000 (0.00%)	0.0042 (−0.43%)	−0.0018 (0.19%)	0.0018 (−0.18%)			
65 or above	0.0000 (0.00%)	0.0005 (−0.06%)	0.0000 (0.00%)	−0.0001 (0.01%)			
**Subtotal**	0.0000 (0.00%)	0.0138 (−1.41%)	−0.0094 (0.96%)	0.0083 (−0.85%)			
**Female**							
Below 15	0.0000 (0.00%)	0.0000 (0.00%)	−0.0050 (0.51%)	0.0000 (0.00%)			
15−24	0.0000 (0.00%)	0.0000 (0.00%)	0.0013 (−0.14%)	0.0016 (−0.16%)			
25−44	0.0036 (−0.37%)	−0.0010 (0.10%)	0.0032 (−0.33%)	−0.0024 (0.25%)			
45−64	−0.0030 (0.30%)	0.0000 (0.00%)	0.0000 (0.00%)	0.0019 (−0.19%)			
65 or above	0.0000 (0.00%)	0.0000 (0.00%)	0.0000 (0.00%)	0.0000 (0.00%)			
**Subtotal**	0.0007 (−0.07%)	−0.0010 (0.10%)	−0.0004 (0.04%)	0.0010 (−0.11%)			
	**Asian, suicide rate per 100,000 people (relative contribution %)**						
	**Drug suicide**	**Firearm suicide**	**Suffocation**	**Other suicide**			
**Male**							
Below 15	0.0000 (0.00%)	0.0000 (0.00%)	−0.0031 (0.32%)	0.0011 (−0.12%)			
15−24	0.0003 (−0.03%)	0.0031 (−0.32%)	−0.0118 (1.21%)	0.0004 (−0.04%)			
25−44	−0.0002 (0.02%)	0.0025 (−0.26%)	0.0019 (−0.20%)	0.0025 (−0.25%)			
45−64	0.0009 (−0.09%)	−0.0027 (0.28%)	−0.0048 (0.49%)	−0.0049 (0.50%)			
65 or above	0.0000 (0.00%)	−0.0004 (0.04%)	−0.0025 (0.26%)	−0.0002 (0.02%)			
**Subtotal**	0.0010 (−0.10%)	0.0025 (−0.26%)	−0.0203 (2.08%)	−0.0011 (0.11%)			
**Female**							
Below 15	0.0000 (0.00%)	0.0000 (0.00%)	0.0000 (0.00%)	0.0000 (0.00%)			
15−24	0.0005 (−0.06%)	−0.0004 (0.04%)	−0.0051 (0.52%)	0.0023 (−0.24%)			
25−44	0.0008 (−0.08%)	−0.0023 (0.23%)	−0.0052 (0.54%)	0.0001 (−0.01%)			
45−64	−0.0027 (0.28%)	−0.0002 (0.02%)	−0.0006 (0.06%)	0.0014 (−0.15%)			
65 or above	0.0045 (−0.46%)	0.0000 (0.00%)	0.0019 (−0.20%)	−0.0002 (0.02%)			
**Subtotal**	0.0031 (−0.32%)	−0.0028 (0.29%)	−0.0090 (0.92%)	0.0037 (−0.38%)			
	**Black/African-American, suicide rate per 100,000 people (relative contribution %)**						
	**Drug suicide**	**Firearm suicide**	**Suffocation**	**Other suicide**			
**Male**							
Below 15	0.0000 (0.00%)	−0.0004 (0.04%)	0.0001 (−0.01%)	0.0000 (0.00%)			
15−24	0.0025 (−0.26%)	0.0308 (−3.15%)	0.0046 (−0.47%)	−0.0002 (0.02%)			
25−44	−0.0026 (0.27%)	0.0427 (−4.37%)	−0.0041 (0.42%)	−0.0039 (0.40%)			
45−64	−0.0006 (0.06%)	0.0004 (−0.04%)	−0.0081 (0.83%)	−0.0010 (0.10%)			
65 or above	0.0000 (0.00%)	0.0004 (−0.04%)	0.0017 (−0.17%)	−0.0009 (0.09%)			
**Subtotal**	−0.0007 (0.07%)	0.0738 (−7.55%)	−0.0057 (0.59%)	−0.0059 (0.60%)			
**Female**							
Below 15	0.0000 (0.00%)	0.0000 (0.00%)	0.0023 (−0.24%)	0.0012 (−0.12%)			
15−24	0.0025 (−0.25%)	0.0017 (−0.17%)	0.0029 (−0.30%)	−0.0005 (0.05%)			
25−44	−0.0010 (0.10%)	0.0045 (−0.46%)	−0.0046 (0.47%)	0.0002 (−0.02%)			
45−64	−0.0019 (0.19%)	−0.0018 (0.19%)	−0.0030 (0.31%)	−0.0024 (0.25%)			
65 or above	0.0000 (0.00%)	0.0004 (−0.04%)	0.0000 (0.00%)	−0.0019 (0.20%)			
**Subtotal**	−0.0004 (0.04%)	0.0047 (−0.48%)	−0.0024 (0.24%)	−0.0035 (0.36%)			
	**HI Native/Pacific Islander, suicide rate per 100,000 people (relative contribution %)**						
	**Drug suicide**	**Firearm suicide**	**Suffocation**	**Other suicide**			
**Male**							
Below 15	0.0000 (0.00%)	0.0000 (0.00%)	0.0000 (0.00%)	0.0000 (0.00%)			
15−24	0.0000 (0.00%)	0.0000 (0.00%)	0.0000 (0.00%)	0.0009 (−0.09%)			
25−44	−0.0031 (0.32%)	−0.0031 (0.32%)	0.0010 (−0.10%)	0.0011 (−0.12%)			
45−64	0.0000 (0.00%)	0.0000 (0.00%)	0.0000 (0.00%)	0.0042 (−0.43%)			
65 or above	0.0000 (0.00%)	0.0000 (0.00%)	0.0000 (0.00%)	0.0000 (0.00%)			
**Subtotal**	−0.0031 (0.32%)	−0.0031 (0.32%)	0.0010 (−0.10%)	0.0063 (−0.64%)			
**Female**							
Below 15	0.0000 (0.00%)	0.0000 (0.00%)	0.0000 (0.00%)	0.0000 (0.00%)			
15−24	0.0000 (0.00%)	0.0000 (0.00%)	0.0000 (0.00%)	0.0000 (0.00%)			
25−44	0.0000 (0.00%)	0.0000 (0.00%)	0.0000 (0.00%)	0.0000 (0.00%)			
45−64	0.0000 (0.00%)	0.0000 (0.00%)	0.0000 (0.00%)	0.0000 (0.00%)			
65 or above	0.0000 (0.00%)	0.0000 (0.00%)	0.0000 (0.00%)	0.0000 (0.00%)			
**Subtotal**	0.0000 (0.00%)	0.0000 (0.00%)	0.0000 (0.00%)	0.0000 (0.00%)			
	**Hispanic, suicide rate per 100,000 people (relative contribution %)**						
	**Drug suicide**	**Firearm suicide**	**Suffocation**	**Other suicide**			
**Male**							
Below 15	0.0000 (0.00%)	0.0026 (−0.28%)	0.0000 (0.00%)	−0.0003 (0.03%)			
15−24	0.0007 (−0.07%)	0.0097 (−1.06%)	−0.0094 (1.02%)	0.0008 (−0.09%)			
25−44	0.0086 (−0.88%)	0.0282 (−3.07%)	0.0133 (−1.45%)	0.0011 (−0.11%)			
45−64	0.0010 (−0.10%)	−0.0131 (1.43%)	−0.0235 (2.55%)	−0.0054 (0.55%)			
65 or above	−0.0028 (0.28%)	−0.0048 (0.52%)	0.0004 (−0.04%)	−0.0026 (0.27%)			
**Subtotal**	0.0075 (−0.76%)	0.0227 (−2.46%)	−0.0191 (2.08%)	−0.0064 (0.65%)			
**Female**							
Below 15	0.0000 (0.00%)	0.0000 (0.00%)	0.0016 (−0.17%)	0.0021 (−0.21%)			
15−24	0.0009 (−0.09%)	0.0016 (−0.17%)	0.0034 (−0.37%)	0.0028 (−0.28%)			
25−44	−0.0024 (0.25%)	0.0050 (−0.54%)	−0.0056 (0.60%)	−0.0009 (0.09%)			
45−64	−0.0038 (0.39%)	−0.0028 (0.30%)	−0.0078 (0.85%)	0.0006 (−0.06%)			
65 or above	−0.0007 (0.07%)	0.0030 (−0.33%)	−0.0003 (0.03%)	−0.0031 (0.32%)			
**Subtotal**	−0.0060 (0.62%)	0.0068 (−0.74%)	−0.0086 (0.94%)	0.0015 (−0.15%)			
	**White, suicide rate per 100,000 people (relative contribution %)**						
	**Drug suicide**	**Firearm suicide**	**Suffocation**	**Other suicide**			
**Male**							
Below 15	0.0000 (0.00%)	0.0047 (−0.48%)	−0.0073 (0.75%)	−0.0003 (0.03%)			
15−24	0.0028 (−0.28%)	0.0064 (−0.66%)	−0.0603 (6.16%)	−0.0193 (1.98%)			
25−44	0.0061 (−0.62%)	0.0147 (−1.51%)	−0.0402 (4.11%)	−0.0010 (0.10%)			
45−64	−0.0467 (4.78%)	−0.1871 (19.14%)	−0.1433 (14.66%)	−0.0672 (6.87%)			
65 or above	−0.0079 (0.81%)	−0.0406 (4.16%)	−0.0177 (1.82%)	−0.0042 (0.43%)			
**Subtotal**	−0.0458 (4.69%)	−0.2018 (20.65%)	−0.2688 (27.50%)	−0.0920 (9.41%)			
**Female**							
Below 15	0.0015 (−0.16%)	−0.0032 (0.33%)	−0.0031 (0.32%)	−0.0006 (0.06%)			
15−24	−0.0041 (0.42%)	−0.0031 (0.32%)	−0.0182 (1.87%)	0.0015 (−0.15%)			
25−44	−0.0251 (2.57%)	−0.0025 (0.25%)	−0.0366 (3.75%)	−0.0187 (1.92%)			
45−64	−0.0736 (7.53%)	−0.0565 (5.78%)	−0.0583 (5.96%)	−0.0136 (1.39%)			
65 or above	−0.0192 (1.96%)	−0.0060 (0.62%)	0.0016 (−0.17%)	−0.0002 (0.02%)			
**Subtotal**	−0.1204 (12.32%)	−0.0713 (7.30%)	−0.1147 (11.73%)	−0.0316 (3.23%)			
	**More than one race, suicide rate per 100,000 people (relative contribution %)**				**Overall, suicide rate per 100,000 people (relative contribution %)**		
	**Drug suicide**	**Firearm suicide**	**Suffocation**	**Other suicide**			
**Male**							
Below 15	0.0000 (0.00%)	0.0000 (0.00%)	0.0000 (0.00%)	−0.0002 (0.02%)	−0.0026 (0.27%)		
15−24	0.0000 (0.00%)	−0.0002 (0.02%)	0.0004 (−0.04%)	−0.0003 (0.03%)	−0.0817 (8.35%)		
25−44	−0.0036 (0.37%)	0.0116 (−1.18%)	0.0012 (−0.12%)	−0.0006 (0.06%)	0.0746 (−7.64%)		
45−64	0.0033 (−0.34%)	−0.0017 (0.17%)	0.0006 (−0.06%)	−0.0020 (0.21%)	−0.5462 (55.88%)		
65 or above	0.0000 (0.00%)	−0.0009 (0.1%)	0.0000 (0.00%)	−0.0012 (0.12%)	−0.0097 (0.99%)		
**Subtotal**	−0.0003 (0.03%)	0.0087 (−0.89%)	0.0022 (−0.22%)	−0.0043 (0.44%)	−0.5656 (57.86%)		
**Female**							
Below 15	0.0000 (0.00%)	0.0000 (0.00%)	0.0000 (0.00%)	0.0000 (0.00%)	−0.0025 (0.25%)		
15−24	0.0036 (−0.37%)	0.0033 (−0.34%)	0.0002 (−0.02%)	−0.0024 (0.24%)	−0.0066 (0.67%)		
25−44	0.0015 (−0.16%)	0.0019 (−0.19%)	0.0001 (−0.01%)	0.0001 (−0.01%)	−0.1010 (10.34%)		
45−64	0.0030 (−0.31%)	−0.0008 (0.08%)	−0.0038 (0.38%)	0.0005 (−0.05%)	−0.2813 (28.78%)		
65 or above	0.0000 (0.00%)	0.0000 (0.00%)	0.0000 (0.00%)	0.0000 (0.00%)	−0.0205 (2.09%)		
**Subtotal**	0.0082 (−0.84%)	0.0044 (−0.45%)	−0.0035 (0.36%)	−0.0018 (0.19%)	−0.4119 (42.14%)		

Some cells are assumed to be 0.0000 (0.00%) due to the CDC WONDER would not provide strata with value less than 10 cases.

Despite a marked decrease in the suicide rate among the White population, we observed that firearm suicide rates rose for persons aged 15–44 years among all groups except HI natives/Pacific islanders (reduced 0.3%). The increase in rates among these age groups ranged from 0.3% (Asian) to 8.1% (Black/African American). Persons of Hispanic background (4.8%) and those of more than one race (1.7%) were also notable.

Table 3 shows that from 2020 to 2023 the pattern increasing rates was similar to the period 2001–2018, with firearm suicide among the White population comprising the major contributor: White males accounted for 51.6% of the increment and White females 12.1%, involving persons ages 25 through 65 + years. An increase in firearm suicides was also evident among minority populations, especially 24- to 44-year-old Black/African-American males, with an increase by 14.0%. Of note, we detected an increasing trend in firearm suicide among the Black/African-American men across the three time intervals studied.

**Table 3.** Contribution of age, sex, ethnicity and methods to the increase of the US suicide rate during the period 2020–2023

	Population structure, suicide rate per 100,000 people (relative contribution %)						
	American Indian or Alaska Native	Asian/Native Hawaiian/Pacific Islanders	Black or African American	HI Native/Pacific Islander	Hispanic	White	More than one race
**Male**							
Below 15	−0.0003 (−0.04%)	0.0000 (0.00%)	−0.0006 (−0.07%)	0.0000 (0.00%)	−0.0004 (−0.05%)	−0.0039 (−0.46%)	0.0000 (0.00%)
15−24	0.0000 (−0.01%)	0.0015 (0.18%)	0.0003 (0.03%)	0.0002 (0.02%)	0.0150 (1.77%)	−0.0161 (−1.90%)	0.0027 (0.31%)
25−44	0.0007 (0.08%)	0.0039 (0.46%)	0.0070 (0.83%)	0.0001 (0.01%)	0.0106 (1.25%)	−0.0337 (−3.97%)	0.0062 (0.73%)
45−64	−0.0008 (−0.09%)	0.0039 (0.46%)	−0.0045 (−0.53%)	0.0002 (0.02%)	0.0167 (1.96%)	−0.1822 (−21.48%)	0.0007 (0.08%)
65 or above	0.0010 (0.11%)	0.0066 (0.77%)	0.0064 (0.75%)	0.0000 (0.00%)	0.0131 (1.54%)	0.1314 (15.49%)	0.0016 (0.19%)
**Subtotal**	0.0005 (0.06%)	0.0158 (1.87%)	0.0086 (1.01%)	0.0005 (0.05%)	0.0549 (6.47%)	−0.1046 (−12.32%)	0.0112 (1.32%)
**Female**							
Below 15	0.0000 (0.00%)	0.0000 (0.00%)	−0.0003 (−0.04%)	0.0000 (0.00%)	−0.0002 (−0.03%)	−0.0018 (−0.21%)	0.0000 (0.00%)
15−24	0.0000 (0.00%)	0.0005 (0.06%)	0.0000 (−0.01%)	0.0000 (0.00%)	0.0037 (0.44%)	−0.0039 (−0.46%)	0.0013 (0.15%)
25−44	0.0003 (0.03%)	0.0010 (0.12%)	0.0008 (0.09%)	0.0000 (0.00%)	0.0030 (0.36%)	−0.0078 (−0.92%)	0.0017 (0.20%)
45−64	−0.0002 (−0.03%)	0.0019 (0.23%)	−0.0009 (−0.11%)	0.0000 (0.00%)	0.0031 (0.37%)	−0.0563 (−6.64%)	0.0006 (0.07%)
65 or above	0.0000 (0.00%)	0.0034 (0.4%)	0.0010 (0.12%)	0.0000 (0.00%)	0.0016 (0.19%)	0.0221 (2.60%)	0.0000 (0.00%)
**Subtotal**	0.0000 (0.00%)	0.0068 (0.8%)	0.0005 (0.06%)	0.0000 (0.00%)	0.0113 (1.33%)	−0.0477 (−5.63%)	0.0035 (0.42%)
	**American Indian or Alaska Native, suicide rate per 100,000 people (relative contribution %)**						
	**Drug suicide**	**Firearm suicide**	**Suffocation**	**Other suicide**			
**Male**							
Below 15	0.0000 (0.00%)	0.0000 (0.00%)	0.0000 (0.00%)	−0.0036 (−0.42%)			
15−24	0.0000 (0.00%)	−0.0064 (−0.76%)	−0.0019 (−0.23%)	−0.0012 (−0.14%)			
25−44	0.0039 (0.46%)	0.0070 (0.82%)	−0.0016 (−0.18%)	−0.0023 (−0.27%)			
45−64	0.0000 (0.00%)	0.0015 (0.17%)	−0.0007 (−0.09%)	−0.0014 (−0.16%)			
65 or above	0.0000 (0.00%)	−0.0005 (−0.06%)	0.0000 (0.00%)	−0.0002 (−0.02%)			
**Subtotal**	0.0039 (0.46%)	0.0015 (0.18%)	−0.0042 (−0.50%)	−0.0087 (−1.02%)			
**Female**							
Below 15	0.0000 (0.00%)	0.0000 (0.00%)	0.0000 (0.00%)	0.0000 (0.00%)			
15−24	0.0000 (0.00%)	0.0000 (0.00%)	0.0011 (0.13%)	−0.0018 (−0.22%)			
25−44	−0.0037 (−0.43%)	0.0002 (0.02%)	−0.0003 (−0.04%)	0.0030 (0.35%)			
45−64	0.0030 (0.35%)	0.0000 (0.00%)	0.0000 (0.00%)	−0.0010 (−0.12%)			
65 or above	0.0000 (0.00%)	0.0000 (0.00%)	0.0000 (0.00%)	0.0000 (0.00%)			
**Subtotal**	−0.0007 (−0.08%)	0.0002 (0.02%)	0.0008 (0.1%)	0.0001 (0.01%)			
	**Asian, suicide rate per 100,000 people (relative contribution %)**						
	**Drug suicide**	**Firearm suicide**	**Suffocation**	**Other suicide**			
**Male**							
Below 15	0.0000 (0.00%)	0.0000 (0.00%)	0.0000 (0.00%)	−0.0033 (−0.39%)			
15−24	0.0011 (0.12%)	0.0052 (0.62%)	0.0009 (0.11%)	0.0039 (0.46%)			
25−44	0.0012 (0.14%)	0.0113 (1.33%)	−0.0052 (−0.61%)	−0.0060 (−0.71%)			
45−64	−0.0021 (−0.25%)	0.0046 (0.54%)	−0.0082 (−0.96%)	0.0016 (0.19%)			
65 or above	0.0000 (0.00%)	0.0046 (0.54%)	−0.0026 (−0.30%)	−0.0001 (−0.01%)			
**Subtotal**	0.0002 (0.02%)	0.0258 (3.04%)	−0.0150 (−1.77%)	−0.0039 (−0.46%)			
**Female**							
Below 15	0.0000 (0.00%)	0.0000 (0.00%)	0.0000 (0.00%)	0.0000 (0.00%)			
15−24	0.0011 (0.13%)	−0.0004 (−0.05%)	0.0012 (0.14%)	−0.002 (−0.24%)			
25−44	−0.0006 (−0.07%)	0.0034 (0.40%)	0.0016 (0.18%)	0.0035 (0.41%)			
45−64	0.0015 (0.18%)	−0.0002 (−0.03%)	−0.0018 (−0.21%)	−0.0012 (−0.14%)			
65 or above	−0.0052 (−0.61%)	0.0000 (0.00%)	−0.0046 (−0.55%)	−0.0012 (−0.14%)			
**Subtotal**	−0.0032 (−0.38%)	0.0028 (0.33%)	−0.0036 (−0.43%)	−0.0009 (−0.11%)			
	**Black/African-American, suicide rate per 100,000 people (relative contribution %)**						
	**Drug suicide**	**Firearm suicide**	**Suffocation**	**Other suicide**			
**Male**							
Below 15	0.0000 (0.00%)	0.0019 (0.23%)	−0.0069 (−0.81%)	−0.0006 (−0.07%)			
15−24	−0.0001 (−0.01%)	0.0230 (2.71%)	−0.0080 (−0.94%)	0.0025 (0.29%)			
25−44	0.0032 (0.38%)	0.0580 (6.84%)	0.0009 (0.11%)	0.0049 (0.58%)			
45−64	0.0017 (0.20%)	0.0353 (4.16%)	0.0055 (0.65%)	0.0011 (0.13%)			
65 or above	0.0000 (0.00%)	0.0003 (0.04%)	0.0009 (0.10%)	−0.0004 (−0.05%)			
**Subtotal**	0.0048 (0.57%)	0.1186 (13.98%)	−0.0076 (−0.90%)	0.0075 (0.89%)			
**Female**							
Below 15	0.0000 (0.00%)	0.0000 (0.00%)	−0.0007 (−0.09%)	−0.0011 (−0.13%)			
15−24	0.0044 (0.52%)	0.0026 (0.3%)	−0.0020 (−0.23%)	0.0029 (0.35%)			
25−44	0.0021 (0.25%)	0.0164 (1.94%)	0.0052 (0.62%)	0.0012 (0.14%)			
45−64	−0.004 (−0.47%)	0.0091 (1.07%)	0.0046 (0.54%)	0.0004 (0.05%)			
65 or above	0.0036 (0.42%)	0.0005 (0.05%)	0.0000 (0.00%)	−0.0007 (−0.08%)			
**Subtotal**	0.0061 (0.72%)	0.0286 (3.37%)	0.0071 (0.84%)	0.0027 (0.32%)			
	**HI Native/Pacific Islander, suicide rate per 100,000 people (relative contribution %)**						
	**Drug suicide**	**Firearm suicide**	**Suffocation**	**Other suicide**			
**Male**							
Below 15	0.0000 (0.00%)	0.0000 (0.00%)	0.0000 (0.00%)	0.0000 (0.00%)			
15−24	0.0000 (0.00%)	0.0000 (0.00%)	0.0000 (0.00%)	0.0007 (0.08%)			
25−44	0.0000 (0.00%)	0.0000 (0.00%)	0.0025 (0.30%)	0.0014 (0.17%)			
45−64	0.0000 (0.00%)	0.0036 (0.42%)	0.0000 (0.00%)	−0.0020 (−0.24%)			
65 or above	0.0000 (0.00%)	0.0000 (0.00%)	0.0000 (0.00%)	0.0000 (0.00%)			
**Subtotal**	0.0000 (0.00%)	0.0036 (0.42%)	0.0025 (0.30%)	0.0001 (0.01%)			
**Female**							
Below 15	0.0000 (0.00%)	0.0000 (0.00%)	0.0000 (0.00%)	0.0000 (0.00%)			
15−24	0.0000 (0.00%)	0.0000 (0.00%)	0.0000 (0.00%)	0.0000 (0.00%)			
25−44	0.0000 (0.00%)	0.0000 (0.00%)	0.0000 (0.00%)	0.0042 (0.49%)			
45−64	0.0000 (0.00%)	0.0000 (0.00%)	0.0000 (0.00%)	0.0000 (0.00%)			
65 or above	0.0000 (0.00%)	0.0000 (0.00%)	0.0000 (0.00%)	0.0000 (0.00%)			
**Subtotal**	0.0000 (0.00%)	0.0000 (0.00%)	0.0000 (0.00%)	0.0042 (0.49%)			
	**Hispanic, suicide rate per 100,000 people (relative contribution %)**						
	**Drug suicide**	**Firearm suicide**	**Suffocation**	**Other suicide**			
**Male**							
Below 15	0.0000 (0.00%)	−0.0026 (−0.30%)	−0.0034 (−0.40%)	0.0003 (0.03%)			
15−24	0.0043 (0.51%)	0.0214 (2.52%)	−0.0145 (−1.71%)	0.0017 (0.20%)			
25−44	−0.0025 (−0.29%)	0.0513 (6.05%)	0.0255 (3.00%)	0.0126 (1.49%)			
45−64	−0.0037 (−0.44%)	0.0145 (1.71%)	0.0177 (2.09%)	0.0035 (0.42%)			
65 or above	0.0021 (0.25%)	−0.0024 (−0.29%)	−0.0040 (−0.47%)	−0.0004 (−0.04%)			
**Subtotal**	0.0001 (0.02%)	0.0822 (9.69%)	0.0214 (2.52%)	0.0177 (2.09%)			
**Female**							
Below 15	0.0000 (0.00%)	0.0000 (0.00%)	−0.0029 (−0.34%)	−0.0010 (−0.11%)			
15−24	−0.0018 (−0.21%)	−0.0014 (−0.17%)	−0.0082 (−0.97%)	0.0050 (0.59%)			
25−44	0.0071 (0.84%)	0.0069 (0.82%)	−0.0039 (−0.46%)	0.0030 (0.35%)			
45−64	0.0022 (0.26%)	0.0045 (0.53%)	0.0047 (0.56%)	−0.0013 (−0.15%)			
65 or above	0.0044 (0.52%)	0.0007 (0.09%)	0.0001 (0.02%)	0.0019 (0.23%)			
**Subtotal**	0.0119 (1.41%)	0.0107 (1.27%)	−0.0101 (−1.19%)	0.0077 (0.91%)			
	**White, suicide rate per 100,000 people (relative contribution %)**						
	**Drug suicide**	**Firearm suicide**	**Suffocation**	**Other suicide**			
**Male**							
Below 15	0.0000 (0.00%)	−0.0059 (−0.70%)	−0.0121 (−1.43%)	0.0016 (0.19%)			
15−24	0.0020 (0.24%)	−0.0249 (−2.93%)	−0.0667 (−7.86%)	0.0122 (1.44%)			
25−44	−0.0065 (−0.77%)	0.1404 (16.54%)	−0.0651 (−7.67%)	0.0090 (1.06%)			
45−64	0.0054 (0.63%)	0.2509 (29.57%)	0.0143 (1.69%)	0.0359 (4.23%)			
65 or above	0.0029 (0.34%)	0.0769 (9.06%)	0.0114 (1.35%)	0.0016 (0.18%)			
**Subtotal**	0.0038 (0.45%)	0.4374 (51.55%)	−0.1182 (−13.93%)	0.0603 (7.10%)			
**Female**							
Below 15	0.0026 (0.31%)	−0.0008 (−0.10%)	0.0056 (0.66%)	0.0003 (0.04%)			
15−24	0.0063 (0.74%)	0.0039 (0.46%)	−0.0145 (−1.71%)	0.0005 (0.06%)			
25−44	0.0020 (0.24%)	0.0248 (2.93%)	−0.0140 (−1.65%)	0.0150 (1.77%)			
45−64	0.0426 (5.02%)	0.0400 (4.72%)	0.0009 (0.10%)	0.0038 (0.45%)			
65 or above	0.0300 (3.54%)	0.0348 (4.10%)	−0.0032 (−0.38%)	0.0102 (1.20%)			
**Subtotal**	0.0835 (9.84%)	0.1027 (12.11%)	−0.0252 (−2.97%)	0.0299 (3.52%)			
	**More than one race, suicide rate per 100,000 people (relative contribution %)**				**Overall, suicide rate per 100,000 people (relative contribution %)**		
	**Drug suicide**	**Firearm suicide**	**Suffocation**	**Other suicide**			
**Male**							
Below 15	0.0000 (0.00%)	0.0000 (0.00%)	0.0002 (0.03%)	0.0002 (0.03%)	−0.0394 (−4.65%)		
15−24	0.0000 (0.00%)	0.0022 (0.26%)	0.0022 (0.26%)	0.0022 (0.26%)	−0.0347 (−4.09%)		
25−44	0.0000 (0.00%)	−0.0014 (−0.17%)	−0.0007 (−0.08%)	−0.0004 (−0.04%)	0.2364 (27.87%)		
45−64	−0.0034 (−0.40%)	0.0026 (0.31%)	0.0023 (0.27%)	0.0004 (0.05%)	0.2146 (25.30%)		
65 or above	0.0000 (0.00%)	−0.0007 (−0.08%)	0.0000 (0.00%)	−0.0011 (−0.13%)	0.2484 (29.28%)		
**Subtotal**	−0.0034 (−0.40%)	0.0027 (0.32%)	0.0040 (0.48%)	0.0014 (0.17%)	0.6254 (73.71%)		
**Female**							
Below 15	0.0000 (0.00%)	0.0000 (0.00%)	0.0000 (0.00%)	0.0000 (0.00%)	−0.0003 (−0.03%)		
15−24	−0.0040 (−0.47%)	−0.0036 (−0.43%)	0.0040 (0.47%)	−0.0025 (−0.30%)	−0.0078 (−0.91%)		
25−44	−0.0021 (−0.24%)	0.0009 (0.11%)	−0.0004 (−0.04%)	0.0000 (0.00%)	0.0747 (8.80%)		
45−64	−0.0002 (−0.02%)	−0.0035 (−0.41%)	0.0001 (0.01%)	0.0004 (0.05%)	0.0528 (6.22%)		
65 or above	0.0000 (0.00%)	0.0000 (0.00%)	0.0042 (0.49%)	0.0000 (0.00%)	0.1036 (12.21%)		
**Subtotal**	−0.0062 (−0.73%)	−0.0062 (−0.73%)	0.0079 (0.93%)	−0.0021 (−0.25%)	0.2230 (26.29%)		

Some cells are assumed to be 0.0000 (0.00%) due to the CDC WONDER would not provide strata with value less than 10 cases.

Suffocation suicides showed a decreasing trend beginning in 2018 and continuing until 2023. Despite an overall population downward trend involving suffocation suicides, increases were evident involving people of Hispanic origin, HI natives/Pacific islanders and persons of more than one race male, and American Indian/Alaska Native, Black/African American and persons of more than one race females. Finally, we observed a substantial decrease of drug-related suicides only among White females (12.3%), mostly deaths ages 45 years and above.

It is important to note that in the context of overall rising rates from 2001 to 2023, the decomposition analysis revealed two opposing population dynamics that affected the overall suicide profile in the United States. The population structure columns in all tables indicated that increasing older adult male populations among all population groups contributed to increased suicide counts whereas the increasing older adult female populations had limited effect as older female suicide rates were not significantly different from the younger ones. At the same time, an overall decrease in the White demographic, especially among males, contributed relatively fewer suicide counts.

## Discussion

The decomposition analysis of suicide rates in the United States from 2001 to 2023 reveals complex patterns across different demographic groups and suicide methods. Notably, firearm and drug-related suicide methods have been the major contributors to the change of suicide rates – with a persistent predominance of firearm suicides, particularly among White males and growing contributions from Black/African American men. The consistent rise in rates among the latter is particularly concerning, even as they currently contribute substantially less than their White counterparts. Further study is needed to discern whether there are common or distinctive demographic and epidemiological characteristics (e.g., age, presence of physical and mental illnesses, geographic location, financial or social circumstances) that require tailored preventive and clinical interventions for firearm suicides.

Our findings reinforce the often stated recommendation for targeted interventions addressing safe firearm storage, as well as lawful removal from persons deemed in danger to themselves or others due to their mental condition (Swanson *et al.,* 2024). Increasing the likelihood of safe home storage is especially challenging among persons who own guns purchased due to concerns about personal or home safety, where ready access is deemed a priority (Caine, 2022). Efforts to date in the United States to foster a reduction in suicide firearm fatalities have been yet to succeed (Ramchand, RAND, as is most evident by their continuing contribution to rising rates.

The slight decrease in suicide rates from 2018 to 2020, primarily driven by the 45–64 age group, reflected fewer firearm suicides in 2019 and a drop in other methods in 2020. The early months of the COVID-19 pandemic overwhelmed state and county death investigation systems in the United States, hindering in-depth examination of cases that did not have easily accessible forensic evidence, such as a suicide note, which may have contributed to misclassification of drug-related suicides as “accidents” (Rockett *et al.,* 2018). Whatever contributing factors played a role, the subsequent increase in suicides after 2020 underscores that the decline was short-lived.

Of note, the reported rate of drug-related suicides changed little overall from 2001 to 2023, a time of surging deaths in the United States due to opioids and other drugs. This is a concerning observation, as drug use confers a marked increase in suicide risk (Oquendo and Volkow, 2018). Medical examiners and coroners faced the task of disentangling the manner of death among overdose victims (Stone *et al.,* 2017) – especially daunting when the age-adjusted rate of drug overdose deaths nearly quadrupled from 8.2 per 100,000 in 2002 to 32.6 per 100,000 in 2022, taking more than 107,000 lives that years (Spencer *et al.,* 2024). As misclassification of such deaths can occur in a high proportion of cases – nearing as high as 30% (Rockett *et al.,* 2018) – this problem may have become even more apparent over the course of time.

These findings have significant implications for suicide prevention strategies and policies. They suggest that a one-size-fits-all approach is insufficient and that interventions should be shaped to the needs of specific demographic groups and suicide methods. Programmes and policies to promote safe firearm storage are being promulgated across the United States, including legislation that supports Extreme Risk Protection Orders (ERPOs), and the CDC has funded grants for Comprehensive Suicide Prevention programmes. However, cultural and political resistance to firearm regulations, particularly in regions with high suicide rates, hinders broader adoption of potential prevention measures.

The decomposition analysis with joinpoint regression facilitated the identification of specific strata that contributed to the shifts in suicide rates in suicide rates in the United States during the 21st century. Decomposition allowed us to disentangle changing demographic characteristics of the overall population that affected rates, and changes stratified by age, sex, ethnicity, race, and method.

This study has three limitations. First, we have assumed the missing value (strata with less than 10 suicide cases that are not disclosed in the dataset) to be 0 to ensure the decomposition works, and this small number might also come from the misclassification of these populations, especially in American Indians/Alaska Natives. Although the WISQARS dataset has implemented specific measures to mitigate such issues, interpreting suicide data in minorities requires extra caution. Our results would underreport such cases, but the effect is expected to be small. The second limitation is that the decomposition is based on the point estimation and report from the WISQARS dataset; we cannot provide any statistical inference to test the significance of the change. Lastly, while this study decomposes the overall suicide rate by age, sex, ethnicity, race, and methods, it reveals nothing regarding factors that contribute to increasing and decreasing trends during the periods. Future research should focus on understanding the protective factors that contributed to the decrease in suicide rates among certain groups, as well as the reasons behind the persistent increase in firearm suicides. Additionally, a more granular analysis of regional variations, such as urban-rural disparity and the impact of specific local state policies on suicide rates, could provide valuable insights for prevention efforts.

## Conclusion

The demographic analysis helped to identify the group(s) that contributed in the United States to the changing pattern of suicide from 2001 to 2023. The change of suicide rate among White men and women had the largest impact on changes in overall United States rates from 2001 to 2023 (contributing to 84.04%, 96.83% and 67.67% of the suicide rate change from 2001 to 2018, 2018 to 2020, and 2020 to 2023, respectively), while controlling the effect of population. Taken together, our results highlight a diversity of population-related factors contributing to rising suicide rates in the United States during the 21st century and underscore the need for preventive interventions that address their specific needs. Also, we again observe the central role of firearms in suicides in the United States – a pattern that became more evident during the second half of the 20th century that continues unabated. The United States, as a society and a nation, has yet to address this complex, fatal issue that is pivotal to reducing what should be preventable, premature deaths.

## Supporting information

10.1017/S2045796025100218.sm001 Yip et al. supplementary material Yip et al. supplementary material

## Data Availability

The data comes from the Government official statistics. It can be accessed on https://wisqars.cdc.gov/

## Supplementary material

The supplementary material for this article can be found at https://doi.org/10.1017/S2045796025100218.
