# Supplementary material for: Decomposing the change of suicide rates in the United States 2001–2023
Source: Epidemiol Psychiatr Sci. 2025 Sep 4;34:e46. doi: 10.1017/S2045796025100218 (PMC12450541; doi:10.1017/S2045796025100218)
Supplement: Yip et al. supplementary material [file S2045796025100218sup001.docx]

**Supplemental Online Content**

**eMethods**. Statistical Methods Description

**eTable1.** Joinpoint regression results for the overall US suicide rates.

**eMethods. Statistical Methods Description**

The suicide rates can be expressed by the following formula:

$$R^{t}= \frac{D_{.}^{t}}{P_{.}^{t}}=\sum_{i} \sum_{j} \sum_{k} \sum_{l} \frac{D_{ijkl}^{t}}{P_{ijk}^{t}}*\frac{P_{ijk}^{t}}{P_{.}^{t}}= \sum_{i} \sum_{j} \sum_{k} \sum_{l} r_{ijkl}^{t}*p_{ijk}^{t}$$

where $R^{t}$ refers to the total crude suicide rate at the time $t$; $D_{.}^{t}$means the total number of suicide deaths at the time $t$;$P_{.}^{t}.$ refers to the total population at the time $t$; $D_{ijkl}^{t}$ denotes suicide death at age group $i$ for sex $j$, ethic $k$ with methods $l$at the time $t$ ,and $P_{ijk}^{t},$ is the population at age $i$ for sex $j$ and ethic $k$ at the time $t$ The suicide rate change due to the population change can be decomposed

Correspondingly, $r_{ijkl}^{t}=$ measures the suicide rates at age $i$ for sex $j$, ethic $k$ with methods $l$ at the time $t$, and $p_{ijk}^{t}=\frac{P_{ijk}^{t}}{P_{.}^{t}}$is the population structure with regard to specific $i$ for sex $j$, ethic $k$ at the time $t$ .

The change of suicide rate at two different time point $t_{1}$ and $t_{0}$

$$R^{t_{1}}-R^{t_{0}}=\Delta R=\sum_{i} \sum_{j} \sum_{k} \sum_{l} \Delta r_{ijkl}\times p_{ijk}^{t_{0}}+\sum_{i} \sum_{j} \sum_{k} \sum_{l} \Delta p_{ijk}\times r_{ijkl}^{t_{1}}$$

where $\Delta r_{ijkl}=r_{ijkl}^{t_{1}}-r_{ijkl}^{t_{0}}$, and $\Delta p_{ijk}=p_{ijk}^{t_{1}}-p_{ijk}^{t_{0}}$. The former term $\Delta r_{ijkl}\times p_{ijk}^{t_{0}}$refers to the suicide rate change due to the suicide rate change, and the later term $\Delta p_{ijk}\times r_{ijkl}^{t_{1}}$ is the suicide rate change due to the population change.

Therefore, the total suicide rate can be decomposed into two constituents, i.e., age-specific suicide rate and population age structure.

**eTable1. Joinpoint regression results for the overall US suicide rates.**

Statistical inference is not available at the turning points 2018 and 2020 as there is only one observation (i.e. Year 2019) between these two turning points.

| **Segment** | **Start year** | **End year** | **Suicide rate change per year (SE)** | ***P* Value** | **BIC3** |
| --- | --- | --- | --- | --- | --- |
| 1 | 2001 | 2005 | 0.08 (0.04) | 0.07 | -2.88 |
| 2 | 2005 | 2018 | 0.29 (0.01) | <0.001 |  |
| 3 | 2018 | 2020 | -0.28 (N/A) | N/A |  |
| 4 | 2020 | 2023 | 0.26 (0.08) | 0.009 |  |
